# Mutations in proteasome-related genes are associated with thyroid hemiagenesis

**DOI:** 10.1007/s12020-017-1287-4

**Published:** 2017-04-07

**Authors:** Bartlomiej Budny, Ewelina Szczepanek-Parulska, Tomasz Zemojtel, Witold Szaflarski, Malgorzata Rydzanicz, Joanna Wesoly, Luiza Handschuh, Kosma Wolinski, Katarzyna Piatek, Marek Niedziela, Katarzyna Ziemnicka, Marek Figlerowicz, Maciej Zabel, Marek Ruchala

**Affiliations:** 1 Department of Endocrinology, Metabolism and Internal Medicine, Poznan University of Medical Sciences, Poznan, Poland; 2 European Center for Bioinformatics and Genomics, Institute of Bioorganic Chemistry, Polish Academy of Science, Poznan, Poland; 3 Department of Histology and Embryology, Poznan University of Medical Sciences, Poznan, Poland; 4 Department of Human Molecular Genetics, Institute of Molecular Biology and Biotechnology, Faculty of Biology, Adam Mickiewicz University, Poznan, Poland; 5 Laboratory of High Throughput Technologies, Institute of Molecular Biology and Biotechnology, Faculty of Biology, Adam Mickiewicz University, Poznan, Poland; 6 Department of Hematology and Bone Marrow Transplantation, Poznan University of Medical Sciences, Poznan, Poland; 7 Department of Paediatric Endocrinology and Rheumatology, Poznan University of Medical Sciences, Poznan, Poland; 8 Institute of Computing Science, Poznan University of Technology, Poznan, Poland

**Keywords:** Thyroid hemiagenesis, Microarray, Exome sequencing, Thyroid transcription factors, Proteasome

## Abstract

**Purpose:**

Human thyroid development is a complex and still unexplained process. Thyroid hemiagenesis is a congenital anomaly, where one of the thyroid lobes fails to develop. In the majority of patients with thyroid hemiagenesis, the genetic background remains unknown. The aim of the study was to search for novel genetic contributors to the etiology of thyroid hemiagenesis.

**Methods:**

A cohort of 34 sporadic patients diagnosed with thyroid hemiagenesis and one three-generation family were subjected to comprehensive genomic examination. Initially, targeted screening of associated transcription factors, known to be linked to thyroid development, was performed. As a next step, genomic examinations were applied using high-resolution microarrays, whereas for the thyroid hemiagenesis family, additionally the whole exome sequencing was performed.

**Results:**

Screening of transcription factors revealed no causative mutations in the studied cohort. Genomic examinations revealed the presence of four recurrent defects (three deletions and one duplication) affecting highly conservative proteasome genes *PSMA1*, *PSMA3*, and *PSMD3*. In a thyroid hemiagenesis family a splice site mutation in a proteasome gene *PSMD2* (c.612T > C cDNA.1170T > C, g.3271T > C) was found in both affected mother and daughter.

**Conclusions:**

Our results shed a new light on etiology of thyroid hemiagenesis, so far suspected to be linked only to mutations in the genes directly involved in the thyroid development. We demonstrated, for the first time, that genomic alterations in proteasome-associated genes co-occur in patients presenting this developmental anomaly.

## Introduction

Thyroid hemiagenesis (THA) is a rare congenital anomaly occurring when one of the thyroid lobes fails to develop. The incidence of the disorder is estimated at 0.05–0.5% of the general population. THA occurs usually as an isolated feature, more frequently in women than in men [1]. THA belongs to the broad and heterogeneous spectrum of thyroid dysgenesis, but in contrast to the other more severe forms, clinical consequences of abnormality rarely include congenital hypothyroidism [2]. The accurate etiology of THA remains unknown, even though the abnormality was found to be an inherited condition in a few families, signifying its genetic origin [3]. Additionally, THA family members commonly present other thyroid developmental anomalies (i.e., thyroid agenesis, ectopy or thyroglossal duct cyst) [4]. This, in turn, suggests common genetic background of different thyroid developmental anomalies, but also contribution of other factors modulating expressivity and severity of the definitive phenotype. The genes known to regulate thyroid embryogenesis (i.e., *NKX2-1*, *FOXE1, PAX8*) are rarely altered in THA patients, and only one THA familial case was reported to be caused by a heterozygous mutation in *PAX8* [5]. Valuable insights into the etiology of the condition provide genetic syndromes, where THA appears as a part of complex phenotype. THA is occasionally concomitant with Williams and DiGeorge syndromes [6]. In both disorders the responsible altered genetic regions are bearing *SHH* and *TBX* genes. Their mutated mouse orthologues were evidenced to cause THA in an animal model [7]. *TBX1* knockout mice presented normal development of follicular cells, whereas due to disturbed formation of ultimobranchial bodies the gland lacked C cells [7]. The animal THA model however, does not reflect accurately the human species, because in surgical specimens obtained from the THA patients the presence of normal C cells was demonstrated [8]. Human *SHH* gene is responsible for severe developmental conditions like holoprosencephaly, and there are no evidences of *SHH* allelism, that may lead to milder phenotype [9]. Other clues coming from THA mouse knockouts encouraged researchers to pay particular attention on human homeobox gene family. The *Hoxa3* null mice particularly show diminished number of follicular cells, hypoplasia or absence of one thyroid lobe which corresponds to human THA [10]. In the recent study by Kizys et al. *HOXA3*, *HOXB3*, *HOXD3*, and *PITX2* genes were analyzed in THA, however no causative mutations were found [11].

Lack of success in identification of common genetic mechanism in THA indicate the need to search for other genetic targets. In this study, we aimed to look for novel genetic factors that contribute to the development of THA in a uniquely large cohort of patients.

## Patients and methods

The studied group consisted of 34 unrelated patients and two familial cases, with THA as an inherited condition transmitted from mother to daughter. The diagnosis of THA was confirmed by ultrasound examination and scintiscan. Recruited patients did not present any concomitant congenital developmental disorders. DNA of all affected individuals and first-degree relatives in THA family was obtained from blood samples. A population cohort of 100 individuals originating from the same region of Poland was used as a control group.

### Genetic examinations

Firstly, we analyzed the genes which role in the thyroid development was documented in previous studies. Sequencing of *PAX8* (NM_003466), *TTF1* (*NKX2-1*, NM_001079668), *TTF2* (*FOXE1*, NM_004473), *HHEX* (NM_002729), *SHH* (NM_000193), and *TBX1* (NM_080647) genes encompassed coding sequences, with neighboring intronic regions. Microarray analyses was performed using Illumina Infinium Human OmniExpress-12v1.0 beadchip (San Diego, USA) and then for selected samples on Affymetrix CytoScanHD arrays (2,7M, Santa Clara, USA). For assays, 200–250 ng of genomic DNA was used. Array scans were managed using Illumina GenomeStudio and Affymetrix Chromosome Analysis Suite (ChAS). Log2 R ratios and B-allele frequencies were used for copy number variations (CNVs) identification in all samples. Population specific CNVs were filtered out (control cohort of 100 healthy individuals were analyzed previously using CytoScan 750 K arrays and included in Poznan University of Medical Sciences database) and then identified abnormalities were compared with CNVs annotated in the database of genomic variants. Findings were validated using Real Time qPCR (ΔΔCT method, Life Technologies). Complete genomic coordinates regarding THA patients were deposited in ClinVar database (Table 1). The whole exome analysis was conducted in a three-generation family, presenting transmission of THA as an isolated feature (pedigree presented on Supplementary Fig. 2). Four family members were examined (proband and their first-degree relatives). A total of 1 μg of genomic DNA from subjects was used for the construction of a library with the TruSeq DNA Sample Preparation Kit (Illumina). Whole exome enrichment was performed with the use of DNA library and TruSeq Exome Enrichment Kit (Illumina). For pathogenicity evaluation, the following features were applied: (a) gene/transcript annotations (downloaded from UCSC GenomeBrowser, hg18), (b) known sequence variants from dbSNP (version 132), 1000 Genomes project (The 1000 Genomes Project Consortium). NGS findings were validated using Sanger sequencing. Strategy presented on Supplementary Fig. 1.

**Table 1:** List of recurrent genomic locations identified in sporadic THA patients

No.	Patient ID	Abnormality type	Chromosome	Position; Start (nt).	Position; End (nt).	Genomic span; Size (Kbp)	ClinVar Accession Number	Genes	No. of reported CNVs/cohort size	Study	*P* value
1.	40 [10]	Duplication	2	45,453,858	45,455,897	2039	SCV000300389	LINC01121	5/771	Pinto et al. [26]	*P* = 0.031475
	32 [3]	Duplication	2	45,454,554	45,457,111	2557	SCV000300390		1/17,421	Cooper et al. 2011[27]	*P* = 0.000011
2.	32 [3]^a^	Deletion	9	107,554,745	110,762,725	3,207,980	–	SLC44A1	1/2026	Shaikh et al. [28]	*P* = 0.000
								FSD1L			785
								FKTN			
								TMEM38B			
								RAD23B			
3.	29 [12]	Deletion	11	14,504,463	14,909,461	404,998	SCV000300391	PSMA1, PDE3B, CYP2R1	1/2504	1000 Genomes Consortium Phase 3[29]	*P* = 0.000518
	28 [11]	Deletion	11	14,657,389	14,918,308	260,919	SCV000300392		1/2026	Shaikh et al. [28]	*P* = 0.000785
4.	11 [6]	Deletion	14	58,737,402	58,884,615	147,213	SCV000300393	PSMA3, ARID4A, TOMM20L, TIMM9	0/17421	Cooper et al. [27]	*P* = 0.000004
	16 [12]	Deletion	14	58,737,402	58,891,576	154,174	SCV000300394				
5.	29 [12]	Deletion	15	62,128,861	62,340,126	211,265	SCV000300395	VPS13C	6/17,421	Cooper et al. [27]	*P* = 0.000102
	13 [10]	Deletion	15	62,155,282	62,332,980	177,698	SCV000300396		1/2026	Shaikh et al. [28]	*P* = 0.000785
							SCV000300397		2/1557	Itsara et al. [30]	*P* = 0.002584
6.	23 [7]^a^	Deletion	17	38,146,929	38,153,473	6544	SCV000300391	PSMD3	0/17,421	Cooper et al. [27]	*P* = 0.000004

^a^ Deletions related to proteasome pathway, but found only in one sporadic patient

#### Prediction of mutations impact and protein network analysis

The identified mutations were analyzed for their impact on protein conformation and functionality. The amino acid changes were examined using Sift [12], and PolyPhen2 [13] as well as Splice Site mutations using Human Splicing Finder (HSF3.0) [14], and MutationTaster [15]. CNVs encompassing genes, were subjected to a search for phenotype relevancy using GENECODIS, whereas functional annotation was conducted with the database for annotation, visualization, and integrated discovery [16]. *P*-values and numerical scores were calculated to rank networks according to their degree of relevance in regards to the different gene lists.

## Results

Sequencing of known thyroid transcription factor genes (*PAX8*, *NKX2-1, FOXE1*, and *HHEX*) and selected targets, demonstrated in previous studies to contribute to the thyroid development (*TBX1*, *SHH*) did not reveal mutations in THA patients. This prompted us to look for genomic abnormalities across sporadic patients, as well as one THA family. The examination using microarrays revealed CNVs (ranging in size from 5 Kb up to 3.2 Mb). We focused particularly on cytoregions bearing genes emerging 63 unique deleterious CNVs. Three deletions turned out to be found in more than one sporadic patient. Analogous investigation was conducted for duplications and selection of 18 gains in copy number were established. Within these loci, a total number of 24 genes were identified and one duplication was found in two unrelated patients. In total four recurrent CNVs (three deletions, one duplication) were detected. We explored DGV to find overlaps with CNVs reported in large control populations, and we found an ultra-rare variability for three out of four regions (not present for *PSMD3* gene, where point mutation in THA family was identified). All four regions were statistically significant and not coincidently enriched comparing to large control populations. Microarray data are summarized in Table 1. Recurrent deletions were harboring highly conservative proteasome genes *PSMA1* (NM_002786, two patients), *PSMA3* (NM_002788, two patients) and additionally deletion encompassing *PSMD3* gene (NM_002809, found in one sporadic patient). In the studied group of patients, deletions of two other proteasome-associated genes *VPS13C* (NM_020821, two patients) and *RAD23B* (NM_002874, one patient) were detected.

**Fig. 1:**
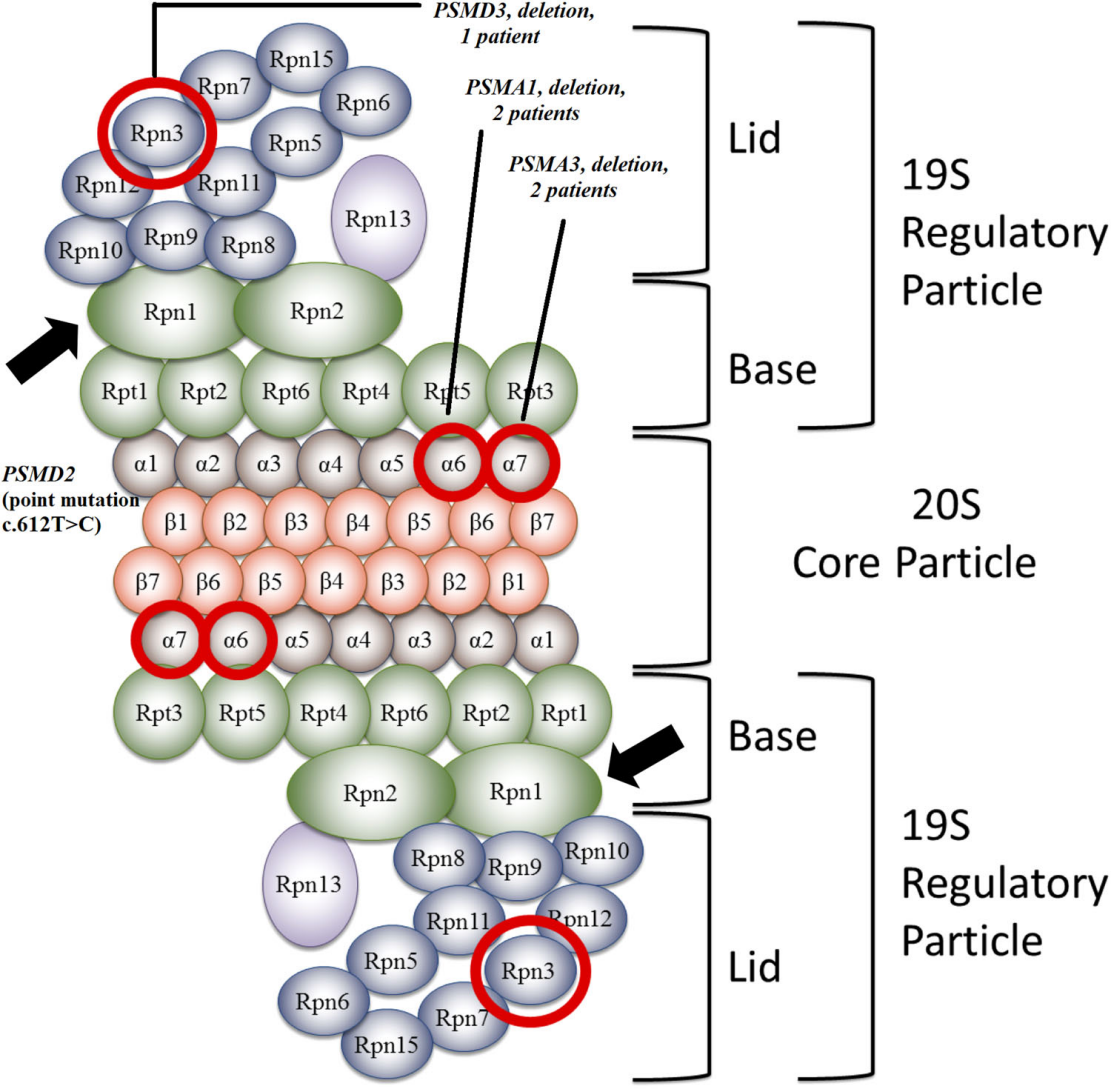
Proteasome and protein units shown to be altered in THA sporadic patients (microarray studies, encircled in *red*) and familial case (WES, an *arrow*). The corresponding gene names as follow: Rpn1—*PSMD2*, Rpn3— *PSMD3*, α6—*PSMA1*, α7—*PSMA3*

Exome-wide sequencing in a THA family resulted in identification of 93 alterations. One change, was a splice site mutation in a proteasome gene *PSMD2* (c.612 T > C cDNA.1170 T > C, g.3271 T > C, NM_002808) found in affected mother and daughter, and regarding microarray findings it was particularly interesting. The mutation introduced an exonic splice sequence (silencer motif), knocking out one *PSMD2* allele. Both microarray and whole exome sequencing (WES) data were concordant and subsequently indicating haploinsufficiency of core proteasome genes (Fig. 1).

Recurrent abnormalities were found in 7 out of 34 patients (20%), and therefore an attempt was made to emerge THA pathways, based on functional interactions between other detected genes/regions. We used all identified CNVs (63 deleterious, 18 gains), bearing in total 175 known genes. This examination resulted in identification of four significant clusters: (a) protein degradation via proteasome, (b) transmembrane transport (solute carriers), (c) cytoskeleton maintenance (actin related) and (d) transcriptional regulation, respectively (Supplementary Table 1). Except proteasome-related genes, none of the above genes altered in more than one patient.

## Discussion

The development of thyroid gland in vertebrates is a multistep complex process. Recently, a comprehensive review summarizing the contribution of transcription factors in thyroid embryogenesis and their role in the differentiation of thyroid follicular cells was published [17]. Mutations of these genes are associated with broad spectrum of clinical features i.e., thyroid hypoplasia, ectopy, agenesis or even syndromal phenotypes characterized by multiple organ development failure. Although the knowledge of the mechanisms involved in the thyroid development has been expanded, in a substantial portion of patients with thyroid dysgenesis it was not possible to identify causative mutations. This suggests that other unknown genes may contribute to the disorder. The aim of the present study was to search for genetic abnormalities explaining the origin of thyroid bilobation failure in humans presenting as an isolated condition.

The transcription factors (TTFs) (*PAX8, FOXE1, HHEX* or, *NKX2-1*) represent different classes of conservative transcription factors. They show expression across various tissues, but appear together exclusively in the thyroid. Their interaction is crucial for proper organogenesis, and single TTF perturbation is leading to severe dysgenesis [18]. In order to identify THA genetic background, a cohort THA patients and a THA family were screened for mutations in TTFs. This examination failed to find any abnormalities in known thyroid genes in THA patients under study. This supports previous findings, that patients with isolated THA in majority do not present mutations in TTFs [4]. Therefore, we searched for defects in other genes that would affect thyroid bud migration, or stabilization of the bilobar thyroid structure. We identified recurrent abnormalities affecting proteasome genes comprising recently an attractive target for the studies on developmental mechanisms. The major function of proteasomes in the cell is degradation of ubiquitinated proteins. Their functional insufficiency leads to accumulation of undegraded protein aggregates, which may exert a toxic effect on the cell. Mutations in proteasome related genes are linked to a broad spectrum of phenotypes encompassing neurological, autoimmune, and developmental disorders [19–21]. We found recurrent deletions of proteasome genes, accounting for portion of patients, that nevertheless is unlikely coincidental. All of the genes—*PSMA1*, *PSMA3* and *PSMD3* as well as *PSMD2* gene in familial case present haploinsufficiency. None of those genes has been linked to the human disease so far. The detected c.612T > C mutation in a *PSMD2* gene, which is transmitted together with a THA in a familial case, introduces an exonic splicing silencer sequence. Remarkably, all the identified genes are coding proteins that orchestrates proteasome, although they represent different functional subunits (*PSMA1*, *PSMA3*—core particle and *PSMD3* as well as *PSMD2*—regulatory particles), depicted on Fig. 1. The proteasome-related pathomechanism was additionally supported by deletions of other proteasome associated genes *VPS13C* (two patients) and *RAD23B* (one patient).

The development of the thyroid is indispensably linked to the cardiovascular system due to juxtaposition of thyroid bud and cardiogenic mesoderm. This fact is supported by high occurrence of heart defects in children with congenital hypothyroidism [22]. On the other hand, the dysfunction of proteasomal-ubiquitin system is recently thoroughly explored in the context of cardiac diseases [23, 24].

Heritability of thyroid dysgenesis accounts for only about 2% of patients and for those cases, the contribution of monogenic background is evidenced (TTFs). So far none of the hypotheses proposed by the Abramowicz et al. (epigenetic regulation, early somatic mutation, stochastic events) referring to missing causes of thyroid developmental anomalies including THA were evidenced [25]. Taking into account high variability and a high frequency of de novo occurring genomic events (particularly CNVs), the model of autosomal dominant transmission proposed by the Macchia et al. is still valid and feasible [5].

In conclusion, our results shed a new light on etiology of THA, so far suspected to be linked only to mutations in the genes directly involved in thyroid development, like TTFs. We demonstrated, for the first time, that genomic alterations in proteasome-associated genes co-occur in patients presenting this developmental anomaly.

## Electronic supplementary material

Supplementary Information

Supplementary Information

Supplementary Information
