# Supplementary material for: Mutations in proteasome-related genes are associated with thyroid hemiagenesis
Source: Endocrine. 2017 Apr 7;56(2):279–85. doi: 10.1007/s12020-017-1287-4 (PMC5395596; doi:10.1007/s12020-017-1287-4)
Supplement: Supplementary file 1 — Supplementary Information [file 12020_2017_1287_MOESM1_ESM.docx]

**Supplementary Figure 1**

Strategy of THA patients examination using conventional approaches (capillary sequencing, MLPA) and high-throughput genomic methods (microarrays, NGS sequencing).

**Supplementary Figure 2**

Abbreviated pedigree of THA family. Affected individuals are presented as blackened symbols. All examined family members (WES analysis) are depicted by an arrow.

**Supplementary Table 1. List of a four gene clusters identified using functional annotation approach using DAVID tool.**

| Gene group 1, Enrichment Score: 1.25 | | |
| --- | --- | --- |
| 1 | CDK1 | [cell division cycle 2, G1 to S and G2 to M](http://david.abcc.ncifcrf.gov/geneReportFull.jsp?rowids=815142) |
| 2 | PSMD3 | [proteasome (prosome, macropain) 26S subunit, non-ATPase, 3](http://david.abcc.ncifcrf.gov/geneReportFull.jsp?rowids=791833) |
| 3 | PSMA3 | [proteasome (prosome, macropain) subunit, alpha type, 3](http://david.abcc.ncifcrf.gov/geneReportFull.jsp?rowids=790104) |
| 4 | PSMA1 | [proteasome (prosome, macropain) subunit, alpha type, 1](http://david.abcc.ncifcrf.gov/geneReportFull.jsp?rowids=805348) |
| Gene group 2, Enrichment Score: 1.05 | | |
| 1 | TMED5 | [transmembrane emp24 protein transport domain containing 5](http://david.abcc.ncifcrf.gov/geneReportFull.jsp?rowids=825703) |
| 2 | C14orf37 | [chromosome 14 open reading frame 37](http://david.abcc.ncifcrf.gov/geneReportFull.jsp?rowids=776190) |
| 3 | SLC23A3 | [solute carrier family 23 (nucleobase transporters), member 3](http://david.abcc.ncifcrf.gov/geneReportFull.jsp?rowids=814448) |
| 4 | TMEM99 | [transmembrane protein 99](http://david.abcc.ncifcrf.gov/geneReportFull.jsp?rowids=809482) |
| 5 | SLCO1C1 | [solute carrier organic anion transporter family, member 1C1](http://david.abcc.ncifcrf.gov/geneReportFull.jsp?rowids=804365) |
| 6 | ELOVL7 | [ELOVL family member 7, elongation of long chain fatty acids (yeast)](http://david.abcc.ncifcrf.gov/geneReportFull.jsp?rowids=803671) |
| Gene group 3, Enrichment Score: 0.9 | | |
| 1 | ACTL7A | [actin-like 7A](http://david.abcc.ncifcrf.gov/geneReportFull.jsp?rowids=819235) |
| 2 | CTNNAL1 | [catenin (cadherin-associated protein), alpha-like 1](http://david.abcc.ncifcrf.gov/geneReportFull.jsp?rowids=823254) |
| 3 | EPB41L4B | [erythrocyte membrane protein band 4.1 like 4B](http://david.abcc.ncifcrf.gov/geneReportFull.jsp?rowids=784809) |
| 4 | ACTL7B | [actin-like 7B](http://david.abcc.ncifcrf.gov/geneReportFull.jsp?rowids=813890) |
| Gene group 4, Enrichment Score: 0.61 | | |
| 1 | ZHX1 | [zinc fingers and homeoboxes 1](http://david.abcc.ncifcrf.gov/geneReportFull.jsp?rowids=817327) |
| 2 | ZEB1 | [zinc finger E-box binding homeobox 1](http://david.abcc.ncifcrf.gov/geneReportFull.jsp?rowids=788323) |
| 3 | KLF4 | [Kruppel-like factor 4 (gut)](http://david.abcc.ncifcrf.gov/geneReportFull.jsp?rowids=802434) |
| 4 | ZNF462 | [zinc finger protein 462](http://david.abcc.ncifcrf.gov/geneReportFull.jsp?rowids=785023) |
| 5 | ZNF682 | [zinc finger protein 682](http://david.abcc.ncifcrf.gov/geneReportFull.jsp?rowids=803624) |
| 6 | ZNF311 | [zinc finger protein 311](http://david.abcc.ncifcrf.gov/geneReportFull.jsp?rowids=821541) |
| 8 | DR1 | [down-regulator of transcription 1, TBP-binding (negative cofactor 2)](http://david.abcc.ncifcrf.gov/geneReportFull.jsp?rowids=807325) |
| 9 | ARID4A | [AT rich interactive domain 4A (RBP1-like)](http://david.abcc.ncifcrf.gov/geneReportFull.jsp?rowids=787152) |
